# “Hot” executive functions are comparable across monolingual and bilingual elementary school children: Results from a study with the Iowa Gambling Task

**DOI:** 10.3389/fpsyg.2022.988609

**Published:** 2022-09-06

**Authors:** Susanne Enke, Catherine Gunzenhauser, Verena E. Johann, Julia Karbach, Henrik Saalbach

**Affiliations:** ^1^Faculty of Education, Leipzig University, Leipzig, Saxony, Germany; ^2^Faculty of Education, Leipzig Research Center for Early Child Development, Leipzig University, Leipzig, Saxony, Germany; ^3^Faculty of Education, University of Freiburg, Freiburg im Breisgau, Baden-Württemberg, Germany; ^4^Department of Psychology, University of Koblenz and Landau, Landau, Rhineland-Palatinate, Germany; ^5^Individual Development and Adaptive Education (IDeA), Frankfurt, Hesse, Germany

**Keywords:** bilingual advantage, executive functions (EF), hot EF, Iowa Gambling Task (IGT), pupillometry, mental effort

## Abstract

Past research found performance differences between monolingual and bilingual children in the domain of executive functions (EF). Furthermore, recent studies have reported advantages in processing efficiency or mental effort in bilingual adults and children. These studies mostly focused on the investigation of “cold” EF tasks. Studies including measures of “hot” EF, i.e., tasks operating in an emotionally significant setting, are limited and hence results are inconclusive. In the present study, we extend previous research by investigating performance in a task of the “hot” EF domain by both behavioral data and mental effort *via* pupillary changes during task performance. Seventy-three monolingual and bilingual school children (mean age = 107.23 months, *SD* = 10.26) solved the Iowa Gambling Task in two different conditions. In the standard task, characterized by constant gains and occasional losses, children did not learn to improve their decision-making behavior. In a reversed task version, characterized by constant losses and occasional gains, both monolinguals and bilinguals learned to improve their decision-making behavior over the course of the task. In both versions of the task, children switched choices more often after losses than after gains. Bilinguals switched their choices less often than monolinguals in the reversed task, indicating a slightly more mature decision-making strategy. Mental effort did not differ between monolinguals and bilinguals. Conclusions of these findings for the bilingual advantage assumption will be discussed.

## Introduction

Recent research suggested that bilingual individuals might have advantages in tasks related to executive function (EF; e.g., Adesope et al., 2010; Engel de Abreu et al., 2012; Poarch and van Hell, 2012; Barac et al., 2014). However, critics of this claim counter that performance differences between monolinguals and bilinguals vastly depend on study features such as age of the participants or task characteristics (e.g., Paap et al., 2015; von Bastian et al., 2016; for recent reviews see Antoniou, 2019; Gunnerud et al., 2020; Lowe et al., 2021).

Broadly speaking, EF subsumes the ability to follow adaptive, goal-directed behaviors and to consciously control thoughts and actions (e.g., Zelazo and Müller, 2002; Garon et al., 2008; Karbach and Unger, 2014). Executive function abilities are especially relevant for academic outcomes (Brock et al., 2009), such as achievement (McClelland and Cameron, 2012; Titz and Karbach, 2014) and classroom behavior (McGlamery et al., 2007). Depending on whether EF operates in emotionally neutral or emotionally relevant situations, some authors distinguish “cold” and “hot” EF, respectively (Zelazo and Müller, 2002).

In the literature on the bilingual advantage, studies have mostly focused on “cold” EF tasks (see Barac et al., 2014 for a review). Even though a common basis seems to underlie performance on hot and cold EF tasks and most everyday challenges draw on both cold and hot aspects of EF (see also below), studies addressing EF in emotionally significant situations, referred to as “hot” EF, are scarce. Thus, to provide a full picture of the bilingual advantage debate, more studies including measures of hot EF are needed. In one study contrasting monolingual and bilingual preschoolers, Carlson and Meltzoff (2008) included both classical EF measures, such as the Attention Network Task and the Dimensional Change Card Sort task (DCCS), but also two emotionally significant delay tasks. They found that bilingual children outperformed their monolingual peers on tasks loading highly on a factor tapping conflict inhibition aspects of EF. However, the groups did not differ on tasks loading highly on a “delay” factor. To our knowledge, no study thus far has examined performance in “hot” EF measures in school-aged monolingual and bilingual children.

While cold EF skills develop rapidly during the preschool period (Zelazo et al., 2004; Garon et al., 2008), several studies with monolingual children and adolescents have shown that developmental trajectories might differ between hot and cold EF with hot EF developing later and more gradually (e.g., Prencipe et al., 2011; O’Toole et al., 2018). The elementary school period might hence be of special interest for emerging developmental differences in hot EF between monolingual and bilingual children. Furthermore, the reliance on well-studied EF tasks, both of the “hot” and the “cold” domain, which allows more sensitive testing procedures (Antoniou, 2019), might help to improve comparability between studies and hence clarify some ambiguities in the bilingual advantage debate. In the present study, we therefore investigated performance of monolingual and bilingual elementary school children in a child appropriate version of the Iowa Gambling Task (IGT; Crone and van der Molen, 2004), one of the most studied tasks of the “hot” EF domain (Kerr and Zelazo, 2004; Buelow and Suhr, 2009; Toplak et al., 2010; Zelazo and Carlson, 2012). Some authors have suggested that a neurophysiological research approach might substantially contribute to the bilingual advantage debate (e.g., Gold, 2015; Vaughn et al., 2015). This approach can help to understand the cognitive processes underlying monolinguals’ and bilinguals’ performance on EF tasks independent from merely searching for performance differences (Antoniou, 2019).

In a previous study, for instance, Enke et al. (2022) found that bilingual elementary school children exhibited significantly less mental effort than their monolingual peers while conducting the Tower of London task (Shallice, 1982), a task tapping several EF abilities of the “cold” domain. This effect occurred independently of behavioral performance. Hence, in the current study, we examined the participants’ pupillary responses as an indicator of mental effort while conducting the IGT.

### The bilingual advantage debate in a nutshell

Research on bilingualism and its relationship with cognitive development has undergone several shifts of focus. Until the middle of the 20th century, growing up in a bilingual environment was predominantly seen as detrimental for cognitive and language development (e.g., Saer, 1923; for a review see Hakuta, 1986), because bilingualism was thought to cause cognitive overload in the individual. However, starting with a study by Peal and Lambert (1962), attention shifted to possible performance differences between monolingual and bilingual school children in cognitive tasks measuring verbal and nonverbal intelligence in favor of the bilinguals. Since then, numerous studies have investigated the specificity of the bilingual experience in relation to language proficiency, linguistic processing and the related effects on cognition and neural organization (cp. Bialystok, 2017 for a review). Many of those found that bilingual children performed significantly better than monolinguals in tasks tapping conflict aspects of EF, as measured *via* the Flanker task or flanker like tasks (e.g., Kempert et al., 2011; Yang et al., 2011; Engel de Abreu et al., 2012; Poarch and van Hell, 2012; Kapa and Colombo, 2013; Poarch and Bialystok, 2015; Saalbach et al., 2016). Based on Green’s (1998) model on bilingual language control, the researchers around Ellen Bialystok proposed an approach to explain these findings. It claims that the permanent activation of two linguistic systems and the need to inhibit the nontarget one, might train a domain-general control network (Bialystok et al., 2009). However, others suggested that bilinguals might simply live with the occasional intrusion from the other language (Paap et al., 2019). In the Adaptive Control Hypothesis, Green and Abutalebi (2013) later revised Green’s (1998) model on language control. They propose that language control demands are dependent on the specific (interactional) context of language production and that therefore cognitive processes might be affected differently in each of these contexts.

Some support for those theoretical ideas comes from studies using neurophysiological methodology. The experience of using two languages on a regular basis entails the potential to systematically influence brain structure and connectivity, although these changes seem to depend on duration and onset of dual language exposure (e.g., Li et al., 2014; Pliatsikas, 2020). Studies with both adult and child participants have shown that bilingualism can lead to changes in gray matter volume and density in certain brain structures (e.g., Mechelli et al., 2004; Della Rosa et al., 2013), like the dorsal anterior cingulate cortex (ACC; Abutalebi et al., 2012) that is related to the executive control network, or also to changes in functional neural circuitry (e.g., Grady et al., 2015; Becker et al., 2016). In a recent meta-analysis, Sulpizio et al. (2020) analyzed functional neuroimaging studies on bilingual language processing. They describe that in bilinguals, regions are recruited for language control that have previously been described as responsible for domain general control processes, including prioritization of information and conflict monitoring (Botvinick et al., 2001; Kerns et al., 2004).

To sum up, the existence of a general bilingual advantage is still under debate. During the last years, authors have suggested to move away from the yes/no question (Paap et al., 2016) and that (1) more sensitive testing procedures and (2) the consideration of neurophysiological methods might help clarify the picture (Antoniou, 2019).

### Bilingualism and hot executive functions

The term hot EF was introduced by Zelazo and Müller (2002) to describe processes underlying goal-directed behavior that operate in emotionally and motivationally relevant contexts. While cold EF has been associated with lateral frontal cortex regions, hot EF typically recruits regions of the orbitofrontal and medial cortex (e.g., Happaney et al., 2004). The conceptual clarity of the distinction between hot and cool EF is still under debate (Peterson and Welsh, 2014; Welsh and Peterson, 2014), also because task difficulty is hardly comparable between hot and cold EF tasks. However, some studies with adolescents point to differing developmental trajectories of hot EF tasks, like the IGT, and more purely cognitive, cold EF tasks (Crone and van der Molen, 2004; Hooper et al., 2004; Prencipe et al., 2011). Prencipe et al. (2011) investigated the development of EF in a sample of 102 children between 8 and 15 years of age that completed several cold and hot EF tasks. They found that all EF measures loaded on one single factor. This indicates that a common basis seems to underlie performance on hot and cold EF tasks with hot EF additionally addressing emotional and motivational processes. Relatedly, Meuwissen and Zelazo (2014) have argued that most everyday challenges draw on both cold and hot aspects of EF (see Castillo, 2021). As described above, EF is usually needed to control thoughts and actions in order to make it more likely to attain a certain goal (Zelazo and Müller, 2002; Garon et al., 2008; Karbach and Unger, 2014) – which implies that the individual would not be indifferent to the outcome (see Hofmann et al., 2012). Others have suggested that most EF tasks are best represented by a continuum of cooler and hotter tasks and that a clear distinction is not always possible (Welsh and Peterson, 2014; Castillo, 2021). Furthermore, evidence from neuroimaging studies supports the notion that while recruitment of certain brain circuits differs for hot or cold EF, the identified regions are not functionally limited to either hot or cold cognition and that the distinction depends on factors such as task features (Salehinejad et al., 2021).

While the most widely discussed explanations for a possible bilingual advantage in cold EF (see above) focus on the purely “cold” cognitive processes of inhibiting a non-target linguistic system, it has also been suggested that the communicative challenges imposed by a bilingual environment might also draw on social cognitive skills such as being more attentive to the interlocutors’ nonverbal signals and possible intentions (Yow and Markman, 2011, 2015). These emotionally and motivationally relevant communicative situations might therefore provide unique experiences for bilinguals to train their “cold” and “hot” EF in an integrated way.

On the other hand, even when assuming that the bilingual advantage is primarily an advantage in “cold” EF, it could be argued that this advantage might result in less cognitive effort in situations requiring EF, thus resulting in enhanced cognitive resources for the motivational and emotional aspects of a situation (Barker and Bialystok, 2019). Therefore, it is reasonable to extend research on monolingual and bilingual differences to this domain.

To date, very few studies have investigated performance on hot EF tasks in bilingual children. Some studies have looked at performance differences in monolingual vs. bilingual preschoolers. As described before, Carlson and Meltzoff (2008) investigated the effect of language experience in a battery of EF tasks and found that bilinguals were only advantaged in tasks of the “cold” EF domain. Similarly, two other studies (Poulin-Dubois et al., 2011; Verhagen et al., 2017) tested younger preschool children on a comparable set of EF measures. They did not find any difference between the two language groups in two delay tasks, but only for a Stroop task. In a more recent study, Nayak and Tarullo (2020) compared performance of monolingual and bilingual preschoolers in an emotional neutral and an emotional significant version of the DCCS. Bilinguals showed faster reaction times in the pre-switch trials of the classic DCCS and faster reaction times in the post-switch trials of the emotionally enriched version of the task. No differences appeared regarding accuracy. The authors interpreted this finding as showing that a potential bilingual advantage appears only in tasks of moderate difficulty. Furthermore, EEG data were recorded and showed that bilinguals had smaller error-related negativity (ERN) peak amplitudes on error-trials in the standard DCCS. The ERN originates in the ACC and is typically interpreted in terms of an index for conflict monitoring (e.g., Yeung et al., 2004). Less activity in the ACC at the same performance level has sometimes been interpreted in terms of higher processing efficiency (c.f., Abutalebi et al., 2012). The smaller ERN peak amplitudes in the bilingual group in the study by Nayak and Tarullo (2020), might be an indicator for lower activity in the ACC and, hence, for more efficient neural processing in the context of conflict monitoring (see also below). Prior research on hot EF in monolingual vs. bilingual children has so far only been conducted in the preschool years. As discussed above, developmental research indicates that significant development of hot EF rather occurs during the school years.

In the only study investigating emotionally significant EF in bilingual school children, Janus and Bialystok (2018) studied working memory performance of monolingual and bilingual elementary school children within an emotional context. They found that bilinguals performed significantly better in terms of accuracy in both a neutral condition of a working memory task and the emotional significant conditions with angry and happy faces as distractors. However, bilingual children needed significantly longer to reach higher accuracy in the more challenging 2-back condition. That is, in comparison with the monolingual group, bilinguals were able to maintain a high performance accuracy by slowing down instead of responding as fast as they could. The authors interpreted this finding as an advantage for bilingual children in cognitive flexibility showing better adjustment to the task demands.

All in all, the effect of bilingualism on performance in hot EF tasks and the underlying processes is still unclear. More research is needed, especially for school-aged children. The only study investigating hot EF performance in older children (Janus and Bialystok, 2018) included a task from the domain of cold EF enriched with emotionally significant feedback that has not been validated psychometrically. Thus, it remains unclear which exact cognitive abilities were addressed. To improve comparability between studies, we administered the IGT (Bechara et al., 1994), a well-studied task of the hot EF domain. Hot EF has been investigated *via* both decision-making tasks, such as the IGT, but also *via* delay tasks, such as delay discounting or delay of gratification (see Zelazo and Carlson, 2020). However, the IGT that relies on both cold and hot EF (Zelazo and Carlson, 2020), might be especially suited for addressing the question of a bilingual advantage in hot EF, since comparable processes (such as inhibitory control) might be responsible for good performance (see below). In the IGT, participants have to choose one out of four decks of cards to receive monetary gains. With a certain probability that varies between decks, money or points are gained and lost and participants have to extend their gains by learning to choose the two decks that are most advantageous. Performance on the IGT typically improves with age and the increasing ability to refrain from switching to another option after facing a loss (which occasionally also happens on the more advantageous decks). Thus, immature decision-making is related to the inability to inhibit intuitive exploration of options after losses (cp. Cassotti et al., 2014). Assuming that bilingual school children outperform their monolingual peers in inhibitory control related tasks, they might also show better performance in emotionally significant contexts such as in the IGT. In a recent meta-analysis, Gunnerud et al. (2020) found no evidence for a bilingual advantage in hot EF tasks, as measured *via* gift delay tasks. However, the authors noted that due to a limited number of studies, no clear conclusions can be drawn. Furthermore, diverging results have been found regarding performance in (a) the standard version of the IGT, where participants encounter gains in each trial of the task while sometimes losing points, and in (b) a reversed task version, where participants regularly lose points while sometimes facing gains. Typically, children learn more rapidly to choose advantageous doors in a reversed task version (e.g., Bechara et al., 2000; Crone and van der Molen, 2004, Crone et al., 2005). One explanation is that winning items might affect children’s decision-making more than losing items (Huizenga et al., 2007), and children might therefore be guided by immediate gains while ignoring future prospects (Schlottmann, 2000). Hence, we included both task versions in our study to extend previous research designs to a sample of multilingual children.

### Cognitive processes underlying executive function task performance of monolinguals and bilinguals

Besides investigating mere performance differences between monolinguals and bilinguals, it has been suggested to shed a light on the underlying mechanisms in bilinguals’ cognitive processing (e.g., Valian, 2015). Studies with adult participants have shown that the bilingual experience can lead to advantages in terms of more efficient neural processing (Abutalebi et al., 2012; Gold et al., 2013; Berroir et al., 2017). This effect might be explained by the experience of regularly monitoring and switching languages (Bialystok, 2017). Regular task practice can affect the cognitive operations underlying task performance, i.e., the functional organization of neuronal circuits. Automatized task performance requires less involvement of the prefrontal control network as reflected by substantial decreases of neural activation (Kelly and Garavan, 2005). Bilinguals may thus have an advantage in processing efficiency which may or may not come with advantages in task performance.

In fact, in a study with school-aged children, Enke et al. (2022) investigated the processing efficiency, or mental effort, of monolingual and bilingual participants conducting the Tower of London task (Shallice, 1982). The children solved 18 items in three experimental conditions of different levels of difficulty. In all conditions, the bilinguals showed significantly less effort than their monolingual peers. The level of performance was statistically controlled for, indicating a higher efficiency of cognitive operations. Mental effort in this study was assessed *via* changes in pupil diameter – providing an objective measure for participants who are too young to make reliable self-assessments. The pupillary response is related to the locus coeruleus, a brain structure controlled by the ACC (e.g., Beatty and Lucero-Wagoner, 2000; Aston-Jones and Cohen, 2005). Pupillometry is not only a well-established method for the assessment of mental effort during execution of cognitive tasks (Eckstein et al., 2017; van der Wel and van Steenbergen, 2018), but also a promising method for developmental research as it is much more easily applicable for children than other neurophysiological methods (Eckstein et al., 2017; Bonmassar et al., 2020). The labels to describe the processes associated with pupillary changes differ between theories (Eckstein et al., 2017), and it has also been suggested that pupil dilation reflects capacity utilization (Just et al., 2003) or unexpected uncertainty/surprise (Aston-Jones and Cohen, 2005; Lavín et al., 2013). However, all of these descriptions are related to intensity- and attention-related aspects of cognitive processing (Eckstein et al., 2017) and in the context of enhanced cognitive control, mental effort has been established as the dominant interpretation (van der Wel and van Steenbergen, 2018).

### The present study

In the present study, we pursue three aims. The first aim was to replicate results from previous studies concerning decision-making strategies in a multilingual sample. We expected bilingual and monolingual children to show comparable patterns of improvement in the selection of advantageous vs. disadvantageous options as found in studies with monolingual children of the same age (Crone and van der Molen, 2004; Prencipe et al., 2011). The second aim was to examine whether and to which extent the bilingual advantages frequently found for children on tasks tapping conflict aspects of (“cold”) EF extend to tasks which require EF to operate in emotionally significant situations (“hot” EF tasks). According to Cassotti et al. (2014), immature decision-making can be traced back to difficulties to execute inhibitory control on the tendency to automatically shift responses after a loss. We thus expected bilinguals to show more mature decision-making than their monolingual peers, as indicated by fewer switches after losses. The third aim was to examine mental effort during task execution in a hot EF task. We asked whether bilinguals would exhibit less effort than their monolingual peers. Since effects of cognitive practice on neural organization have only been observed for tasks of the cold EF domain, we were treating this research question as exploratory.

## Materials and methods

### Participants

Seventy-three elementary school children from two large German cities participated in this study (mean age in months = 107.23, *SD* = 10.26, range = 84–131). Participants were either monolingual German speaking (*n* = 38) or bilingual German-Russian speaking (*n* = 35). German-Russian bilinguals were chosen because families with a Russian background form one of the largest bilingual communities at the sites of data collection. Parents and children provided written informed consent and parents completed a questionnaire including questions on sociodemographic information of the family and the child’s language background. Based on this information, children were included in the bilingual group if they had regular contact to both languages starting at the age of three or earlier. No other language besides German and Russian should be spoken by the children. Monolinguals were speakers of German and should not speak any other language (except of those taught in school). One child in the monolingual group had to be excluded from the analyses, because parents indicated that a second language was spoken on a regular basis.

We assessed parents’ highest educational degree as an indicator of socioeconomic status (SES) and calculated the mean value of both parents. Monolinguals and bilinguals did not differ in terms of SES, *t*(70) = 1.23, *p* = 0.22. In our sample, 58% of the parents were qualified to enter universities; the other parents had a secondary (modern) school degree. We also assessed parents’ education after school. In 67% of the families, at least one parent had an academic degree. We compared monolingual and bilingual children on several other background variables. Children neither differed in age, *t*(71) = 0.66, *p* = 0.509, nor in nonverbal intelligence, *t*(60.11) = 1.63, *p* = 0.109 (see also Table 1 and section *control variables*). However, monolinguals outperformed bilinguals in German language abilities [expressive vocabulary: *t*(54.09) = 6.70, *p* < 0.001, *d* = 1.60, grammar understanding: *t*(58.41) = 2.26, *p* = 0.027, *d* = 0.54]. We therefore included measures of vocabulary and grammar understanding as control variables in our analyses, also because the development of EF and linguistic abilities are closely related (e.g., Bohlmann et al., 2015; Cadima et al., 2019). Although we found no differences in nonverbal intelligence across the monolingual and the bilingual group, we also included it as a control variable, because there is evidence for a relation between general intelligence and IGT performance as a measure for “hot” EF (e.g., Gansler et al., 2011b).

**TABLE 1:** Descriptive statistics, results from independent samples *t*-tests and bivariate correlations of main study variables.

	Monolinguals			Bilinguals														
		
Variable	*M*	*SD*	*n*	*M*	*SD*	*n*	*t*	df	*p*	(1)	(2)	(3)	(4)	(5)	(6)	(7)	(8)	(9)
(1) Parents’ highest education^a^	2.71	0.49	38	2.56	0.56	34	1.23	70	0.224	−	0.119	0.379	0.269	0.550**	0.204	−0.505**	–0.013	0.184
(2) Age in months	108.00	10.23	38	106.40	10.37	35	0.66	71	0.509	−0.336*	−	0.417^†^	0.295	0.369	0.366*	0.047	–0.143	–0.204
(3) Nonverbal intelligence^b^	31.76	2.87	38	30.40	4.12	35	1.63	60.11	0.109	0.114	0.321*	−	0.592**	0.657***	0.401*	–0.274	–0.064	0.124
(4) German expressive vocabulary^b^	29.11	4.40	38	19.37	7.48	35	6.70	54.09	< 0.001	0.216	0.002	0.284^†^	−	0.560**	0.284^†^	–0.173	–0.134	–0.062
(5) German grammar understanding^b^	17.55	2.02	38	16.17	3.04	35	2.26	58.41	0.027	0.344*	–0.056	0.261	0.524***	−	0.294^†^	−0.308^†^	–0.028	0.084
(6) Net scores standard task	–4.36	6.06	38	–2.64	5.71	35	–1.24	71	0.218	0.008	–0.028	0.048	–0.041	0.321*	−	−0.583***	0.312^†^	0.267
(7) Net scores reversed task	3.62	4.27	38	2.88	4.62	34	0.71	70	0.483	0.128	–0.006	0.024	–0.168	−0.311^†^	−0.538***	−	−0.287^†^	−0.538*
(8) Overall switching standard task	0.68	0.24	38	0.70	0.23	35	–0.40	71	0.690	0.088	0.109	0.162	0.001	0.399*	0.795***	−0.414**	−	0.590**
(9) Overall switching reversed task	0.80	0.18	38	0.79	0.18	34	0.16	70	0.875	–0.170	0.115	0.028	–0.012	0.218	0.311^†^	−0.781***	0.407*	−

Coefficients above the diagonal show bilingual sample, and coefficients below the diagonal show monolingual sample.

^a^Three-point scale, relating to Germany’s different educational tracks after primary school, from Hauptschule to graduation from Gymnasium (Abitur), with the latter entitling students to continue studying at university. Mean of mothers and fathers.

^b^Raw scores. ^†^p < 0.10, *p < 0.05, **p < 0.01, ***p < 0.001.

### Experimental procedure

Children completed two one-on-one sessions in a laboratory setting. Experimenters were student assistants trained by the authors. In a first session, children’s linguistic abilities, non-verbal intelligence and performance in a planning task were assessed. Those measures were not analyzed in the present study. In a second session, children completed the IGT and another task which was also not analyzed in the present study. The IGT contained two different conditions, a standard version of the task and a reversed version (see measures). The order of task version was counterbalanced between children. The IGT was presented in OpenSesame (OpenSesame 3.2.4; Mathôt et al., 2012), an open source software for experimental tasks. Children were seated at approximately 58 cm away from the monitor (34.5 cm × 19.4 cm, resolution 1,366 × 768 pixels). An eye-tracking unit (Tobii model X120; Tobii Technology, Stockholm, Sweden) positioned below the monitor recorded children’s eye movements and pupil size at a sampling frequency of 120 Hz. During the first experimental session, parents completed the questionnaire described before. Upon completion of both sessions, the children received a 10 € voucher for a toy store and a certificate as gratification.

### Measures

#### Hot executive functions

**FIGURE 1:**
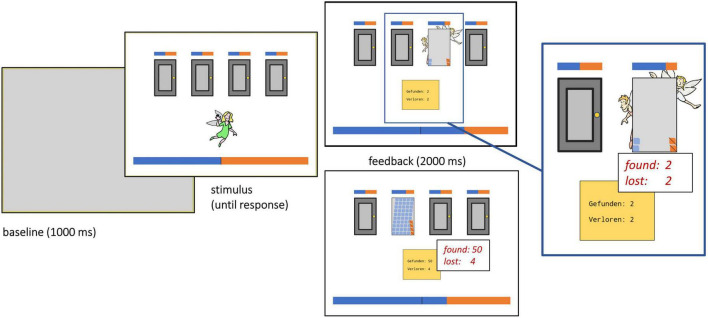
Example of the Iowa Gambling Task as displayed on the screen. After a baseline screen, participants were shown the stimulus screen consisting of four doors and a fairy below the doors. Doors were equal in size. Bars above the doors indicated how many gems had been gained and lost so far for each door following each selection. One large bar at the bottom indicated total gains and losses (for further details on how color distribution was calculated see Crone and van der Molen, 2004). After children had selected one door by clicking on it, the feedback screen was shown for 2,000 ms. The Figure shows two exemplary feedback screens, one for the standard task version (*above*) and one for the reversed task version (*below*).

Hot EF was assessed *via* a modified version of the child friendly IGT developed by Crone and van der Molen (2004) where participants have to help a hungry donkey. This framing is to make the task more meaningful for children. In our task version, children were told to help a fairy gather gems in order to rebuild the fairy’s village that had been hit by a thunderstorm. The gems were hidden behind four different doors. Children were further instructed that sometimes elf children would hide behind the doors playing a trick on the child and trying to take away the gems. Children had to click on one of the four doors that would open and reveal how many gems had been gained or lost. A result screen indicating how many gems had been gained and lost was shown for 2,000 ms (for an illustration of the task set-up, see Figure 1). Like in the study by Crone and van der Molen (2004), children completed two versions of the task with five blocks of 20 trials each resulting in a total number of 200 experimental trials. The relative proportions of gains and losses were distributed according to the original version of the task (Bechara et al., 1994), but absolute amounts were reduced by a factor 25 (see Table 2). In the standard version of the task, all trials resulted in gains with a certain probability of additionally losing gems. Selecting doors A and B would lead to a gain of four gems in each trial. By a probability of 0.5 (door A) and 0.1 (door B), respectively, an additional 10 gems^1^ (door A) or 50 gems (door B) would be lost. This distribution of losses and gains resulted in a net value of 10 lost points over 10 trials. Thus, selecting door A or door B would be disadvantageous on average. Selecting doors C and D would lead to a gain of two gems in each trial. By a probability of 0.5 (door C) and 0.1 (door D), respectively, an additional two gems^2^ (door C) or 10 gems (door D) would be lost. This distribution of losses and gains resulted in a net value of 10 gained points over 10 trials. Thus, selecting door C or door D would be advantageous on average.

**TABLE 2:** Distribution of gains and losses in the standard and the reversed version of the Iowa Gambling Task.

	Standard task				Reversed task			
		
Door	Gain	% Loss	Mean loss	Net score over 10 trials	Loss	% Gain	Mean gain	Net score over 10 trials
A	4	50%	10	−10	4	50%	10	10
B	4	10%	50	−10	4	10%	50	10
C	2	50%	2	10	2	50%	2	−10
D	2	10%	10	10	2	10%	10	−10

In the standard task, participants gained points in each trial while additionally losing points by a certain probability. In the reversed task, participants lost points in each trial while additionally gaining points by a certain probability. Choosing doors A and B would be disadvantageous in the standard task and advantageous in the reversed task. Choosing doors C and D would be advantageous in the standard task and disadvantageous in the reversed task.

In the reversed version of the task, gains and losses were exchanged, meaning that all trials resulted in losses with a certain probability of additionally gaining gems. Selecting doors A and B would lead to a loss of four gems in each trial. By a probability of 0.5 (door A) and 0.1 (door B), respectively, an additional 10 gems (see text footnote 1) (door A) or 50 gems (door B) would be gained. This distribution of losses and gains resulted in a net value of 10 gained points over 10 trials. Thus, in this reversed version of the task, selecting door A or door B would be advantageous on average. Selecting doors C and D would lead to a loss of two gems in each trial. By a probability of 0.5 (door C) and 0.1 (door D), respectively, an additional two gems (see text footnote 2) (door C) or 10 gems (door D) would be gained. This distribution of losses and gains resulted in a net value of 10 lost points over 10 trials. Thus, in this reversed version of the task, selecting door C or door D would be disadvantageous on average.

Past research has reported diverging results regarding the relationship between working memory and IGT performance. In a review by Toplak et al. (2010), only one of fifteen studies found a relationship between working memory and IGT performance. More recent studies using an individual differences approach (Bagneux et al., 2013) or dual task paradigms (Cui et al., 2015) found working memory to be involved in advantageous IGT decision-making. To account for those mixed findings, we therefore applied the approach by Crone and van der Molen (2004) to reduce working memory load during task performance. A bar positioned over each door colored orange and blue indicated how many gems had been gained or lost so far for the respective door. A large bar at the bottom end of the screen contained this information over all choices that had been made (Figure 1; see also Crone and van der Molen, 2004). Two dependent variables were calculated: The net score differences were calculated by taking the difference between the number of advantageous and disadvantageous doors chosen in one block. Net scores could hence reach a minimum value of −20 and a maximum value of 20. Positive scores indicated an overall net gain over trials. We further calculated percentage of switches for each experimental block. This value indicated how often children selected a different door than the door they had chosen in the trial before. Percentage of switches was determined for trials resulting in gains and for trials resulting in losses separately.^3^

#### Mental effort

We assessed pupil dilation as an index of mental effort during IGT performance. At the beginning of task administration, children were guided through a five-point calibration phase. As a first step of data preparation, all data were filtered, interpolated, and averaged across the right and left eyes. Pupil data were filtered by excluding data that differed from the preceding and subsequent samples’ measures by 0.9% (cf. Hepach et al., 2012). Linear interpolation of missing data was applied when the gap between two data points did not exceed four. In each trial, we calculated the relative change in pupil size in relation to the first 100 ms after participants had selected a door, because we were interested in mental effort during processing of the received feedback. We therefore subtracted this baseline value from the average pupil size measured during the feedback screen. This difference score was then divided by the baseline, thus arriving at a baseline-corrected change in pupil size. Because the pupil has an average light adaption of 1.5 s (Eckstein et al., 2017), these first 1.5 s were not included. Pupil dilation was calculated over the five experimental blocks for each of the four doors and for both task versions separately.

#### Control variables

##### Nonverbal intelligence

Nonverbal intelligence was assessed with Raven’s Coloured Progressive Matrices (CPM; Raven et al., 1998). Children have to choose the right one out of six pictures to complete a pattern with a piece missing. The test included 36 items, Cronbach’s alpha in the current sample was α = 0.76.

##### German vocabulary

We assessed German expressive and receptive vocabulary with the *Wortschatz und Wortfindungstest für 6-10-Jährige* (Vocabulary and Word-Finding Test for 6- to 10-Year-Olds, WWT 6-10; Glück, 2011). This standardized test measures expressive vocabulary with a picture naming task of 40 items by showing children pictures of an object, an action or asking them to name the opposite of an adjective. Items not answered correctly by the child are presented again in the second, receptive part of the test where they have to show the right picture out of four. The items answered correctly in the first part are also coded as correctly solved in the second part. Since receptive vocabulary was close to ceiling in both groups of children (mean monolinguals = 39.03, mean bilinguals = 36.26), we included only the measure of expressive vocabulary in our analyses. Cronbach’s alpha for expressive vocabulary was α = 0.91 and α = 0.88 depending on the age appropriate version.

##### German grammar understanding

Grammar understanding was assessed with the German version of the Test for Reception of Grammar (TROG-D; Fox, 2013). Participants hear a sentence of ascending complexity and are then asked to identify a target picture out of a choice of four pictures. In the current sample, Cronbach’s alpha was α = 0.67. We chose to assess grammar understanding in addition to a measure of vocabulary, because previous research on the relation between language and EF has commonly included the assessment of both syntactical and lexical knowledge (e.g., Engel de Abreu, 2011; Saalbach et al., 2016; White et al., 2017).

### Missing values

Missing values in this study were limited. For one participant, data recording did not work during the reversed version of the task, resulting in *n* = 72 for this task version. One participant had no recordings of pupillometry data and was thus excluded from the respective analyses.

### Data preparation and analytic strategy

Data were prepared and analyzed using *R* version 4.1.0 (R Core Team, 2021). The present repeated measures data was hierarchically structured (several observations [level-1] that are nested within persons [level-2]). These data can be analyzed by conventional repeated measures analysis of variance (RM-ANOVA). However, RM-ANOVA entails several disadvantages that can be overcome by a multi-level approach. For instance, MLM does not require the assumption of sphericity or compound symmetry and it has a higher power in hypothesis testing (e.g., Quené and van den Bergh, 2004). Therefore, all hypotheses were tested by means of multi-level modeling (MLM). MLM was conducted with the *lme4* package by Bates et al. (2015).

To address research aims 1 and 2, growth curve models over the five experimental blocks were specified for each task version. Experimental block as a level-1 predictor would thus indicate changes in choice and switching strategies over the course of the task. The level-2 predictor language group indicated whether growth rates would be moderated by this variable. The analysis of switching strategies included a level-1 predictor indicating whether a trial was a gain or a loss trial. The information of a trial being a gain or a loss trial was stored in a dummy coded variable. The variable indicating the experimental block was centered on block 1 which provided a meaningful zero point (cf., Hülsheger et al., 2014). When cross-level interactions are of interest, Enders and Tofighi (2007) recommend centering of variables within level-2 groups instead of grand-mean centering. Hence, we centered net scores in relation to the net scores of the first experimental block within each participant. Percentage of switches after gain and loss trials were centered relative to a switching rate of one hundred percent (i.e., 1) within each participant. Positive parameter estimates thus indicate an increase in switches and negative parameter estimates indicate a decrease in switches. All models specified to answer research questions 1 and 2 included nonverbal intelligence, expressive vocabulary and grammar understanding as level-2 predictors. The third aim was addressed with multilevel models that predicted changes in pupil diameter from the door that was chosen in a trial and language group. Again, models were specified for both task versions separately. Door was added as a level-1 predictor and language group as level-2 predictor. The door chosen was represented by two dummy coded variables, relating to the two factors advantageous vs. disadvantageous doors and frequent vs. occasional gains/losses, respectively. The first variable x_1_ indicated advantageous vs. disadvantageous doors (i.e., x_1_ = 0 for doors A and B vs. x_1_ = 1 for doors C and D). The second variable x_2_ indicated frequent vs. occasional gains/losses (i.e., x_2_ = 0 for doors A and C vs. x_2_ = 1 for doors B and D). Hence, the four doors could be represented unequivocal by those two variables. For the standard task, only trials that resulted in a loss of gems were included in the analyses and for the reversed task, only trials that resulted in a gain were included, because we were interested in the cognitive processes related to deviations from the task’s standard situation (i.e., gains in the standard version and losses in the reversed version). The variable indicating changes in pupil diameter was centered within participants in relation to the score of door A.

All models were fit using full maximum likelihood estimation. Restricted maximum likelihood usually provides less biased estimates (McNeish and Stapleton, 2016). However, the functions in *R* providing model comparisons are only applicable when models are fit with full maximum likelihood. Also, when the number of clusters is greater than 30, full maximum likelihood estimates usually provide reasonable results (McNeish and Stapleton, 2016). Model fits were evaluated *via* Akaike Information Criterion (AIC) and χ^2^-tests that compared models. We further calculated pseudo-*R*^2^ statistics to have an approximation of explained variance (Snijders and Boskers, 1994).

For all research questions, an unconditional random coefficient model was estimated in a first step to have an indicator of the relative amount of between-person and within-person variance *via* intraclass coefficients (*ICC1*). Concerning net scores, 47% of variation in the standard version of the task could be attributed to variation between individuals (level-2 variation). In the reversed version of the task, 49% of variation in nets scores could be attributed to variation between individuals. Concerning percentage of switches, 33% of variation in the standard version of the task could be attributed to variation between individuals. In the reversed version of the task 40% of variation could be attributed to variation between individuals. Concerning pupil dilation, 16% of variation in the standard version of the task could be attributed to variation between individuals. In the reversed version of the task, 21% of variation could be attributed to variation between individuals. Likelihood ratio tests indicated that this level-2 variation was statistically significant (all *p* values < 0.01), hence justifying MLM.

## Results

### Preliminary analyses

In a first step, we tested whether change in net scores in the standard and the reversed version of the task would differ depending on the order of task administration. Therefore, we conducted two independent samples *t*-tests using the difference in net scores between block 5 and block 1 as dependent variable and randomization group as grouping variable. For both the standard and the reversed task, difference in net scores between block 5 and block 1 did not differ depending on whether participants conducted the task first or second, *t*(71) = 0.87, *p* = 0.387 and *t*(71) = −0.25, *p* = 0.805, respectively. We further tested whether change in percentage of switches after gains or losses in the standard and the reversed version of the task would differ depending on the order of task administration. Therefore, we conducted independent samples *t*-tests using the difference in percentage of switches between block 5 and block 1 as dependent variable and randomization group as grouping variable. In both task versions and for both switches after gains and after losses, difference in percentage of switches between block 5 and block 1 did not differ dependent on whether participants conducted the task first or second, all *p*’s > 0.20.

Furthermore, difference in net scores between block 5 and block 1 did not differ between test sites for both task versions, *p*’s > 0.20, nor did the difference in percentage of switches between block 5 and block 1, *p*’s > 0.10.

### Research question 1: Selection of advantageous versus disadvantageous options over the course of the task

We first tested a random intercept, fixed slope model, using block as a predictor of net scores (Table 3, Model 1). In the standard task, no predictor was statistically significant. In the reversed task version, the significant variable experimental block indicated that more advantageous doors were chosen during the course of the task. Grammar understanding was a further significant predictor and indicated that children with better syntactical knowledge chose less advantageous doors on average.

**TABLE 3:** Growth curve models of change trajectories in net scores over blocks.

	Standard task						Reversed task					
		
	Model 1			Model 2			Model 1			Model 2		
				
Variable	Estimate	SE	SD	Estimate	SE	SD	Estimate	SE	SD	Estimate	SE	SD
**Fixed effects**												
Intercept (Block 1)	–8.71	6.33		–3.75	4.30		1.83	3.53		–1.96	3.08	
Block (growth rate)	–0.04	0.21		–0.07	0.40		0.95***	0.14		0.95***	0.23	
Nonverbal intelligence	–0.03	0.24		–0.03	0.16		0.17	0.13		0.18	0.11	
Expressive vocabulary	0.03	0.11		0.02	0.09		0.00	0.06		0.02	0.07	
Grammar understanding	0.50	0.34		0.21	0.23		−0.44*	0.19		–0.28	0.17	
Language group^a^				1.00	1.19					0.72	0.88	
Block × language group				0.08	0.58					0.01	0.33	
**Random effects**												
Intercept			26.77			2.16			7.32			1.21
Block						3.94						0.76
Residual			31.60			21.28			14.22			12.13
**Model fits**												
χ^2^ (*df*)				98.44(*4*)***						33.83(*4*)***		
AIC	2,431.04			2,340.60			2,082.95			2,057.12		
Pseudo-*R*^2^ ^b^	0.03			0.61			0.11			0.45		

^a^Dummy coded: monolingual = 0, bilingual = 1.

^b^Snijders and Boskers (1994). *p < 0.05, ***p < 0.001.

**FIGURE 2:**
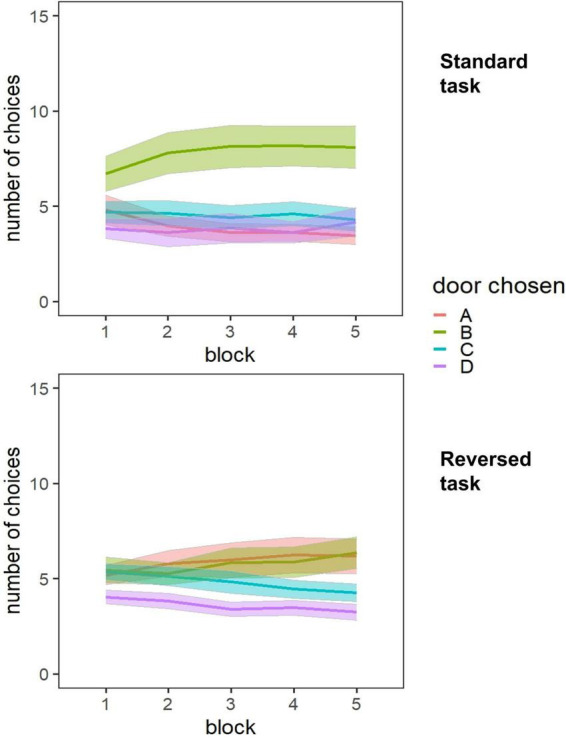
Number of doors chosen as a function of trial block for the standard and the reversed task.

In the second step, we specified a random intercept random slope model (Model 2, final model). This strategy assumed that the relationship between experimental block and doors chosen varies among individuals. In this step, we included language group as a level-2 predictor and the respective interaction term. For the standard task, no predictor reached statistical significance. For the reversed task, block remained a significant predictor. This indicated that both monolingual and bilingual children improved their decision-making behavior over the course of the task. To illustrate decision-making behavior in both task versions in more detail, we plotted the number of doors chosen as a function of trial block for both tasks (see Figure 2). In the standard task, children stuck to choosing the disadvantageous door B over all experimental blocks. In the reversed task, children chose the advantageous doors more and more frequently as the task progressed. For both the standard and the reversed task, model fits improved in the second step.

### Research question 2: Switching after gains and losses

First, we tested a random intercept, fixed slope model, using block as a predictor of percentage of switches (Table 4, Model 1). The dummy coded variable indicating whether switches referred to the mean switches after gain or after loss trials (gain/loss) was added as level-1 predictor. Intercepts indicate the mean deviation from a switching rate of one hundred percent in block 1 in gain trials. We controlled for attained net scores per block by adding the centered level-1 predictor net score. In both the standard and the reversed task, the gain/loss slopes reached statistical significance, indicating more switches in loss as compared to gain trials. Furthermore, net scores were related to switching behavior in both task versions. However, in the standard task the relation was in a positive direction while in the reversed task the relation was negative.

**TABLE 4:** Growth curve models of change trajectories in percentage of switches after gains and losses over blocks.

	Standard task						Reversed task					
		
	Model 1			Model 2			Model 1			Model 2		
				
Variable	Estimate	SE	SD	Estimate	SE	SD	Estimate	SE	SD	Estimate	SE	SD
**Fixed effects**												
Intercept (Block 1)	−0.46**	0.21		–0.38	0.21		−0.50*	0.20		0.64***	0.19	
Block (growth rate)	–0.01	0.01		–0.02	0.01		–0.00	0.01		–0.02	0.01	
Gain/loss^a^	0.21***	0.03		0.22***	0.04		0.18***	0.02		0.15***	0.03	
Net score	0.01***	0.00		0.00**	0.00		−0.01***	0.00		−0.01**	0.00	
Nonverbal intelligence	0.00	0.01		0.00	0.01		0.00	0.01		0.00	0.01	
Expressive vocabulary	–0.01	0.00		–0.01	0.00		–0.00	0.00		−0.01*	0.00	
Grammar understanding	0.01	0.01		0.01	0.01		0.02	0.01		0.01	0.01	
Block × gain/loss	–0.00	0.01		0.01	0.02		–0.01	0.01		–0.00	0.01	
Language group^b^				–0.06	0.07					−0.13*	0.06	
Block × language group				0.02	0.02					0.03	0.02	
Gain/loss × language group				–0.02	0.05					0.06	0.05	
Block × gain/loss × language group				–0.02	0.02					–0.02	0.02	
**Random effects**												
Intercept			0.03			0.03			0.03			0.02
Block						0.00						0.00
Residual			0.05			0.05			0.04			0.03
**Model fits**												
χ^2^ (*df*)				11.94^†^(*6*)						19.96**(*6*)		
AIC	23.33			23.39			−159.85			−167.81		
Pseudo-*R*^2^ ^c^	0.14			0.20			0.12			0.27		

^a^Dummy coded: gain trial = 0, loss trial = 1.

^b^Dummy coded: monolingual = 0, bilingual = 1.

^c^Snijders and Boskers (1994).

^†^p < 0.10, *p < 0.05, **p < 0.01, ***p < 0.001.

In the second step, we specified a random intercept random slope model (Model 2, final model). This assumed that the relationship between experimental block and switching behavior varies among individuals. In this step we included language group as a level-2 predictor and the respective interaction terms with the level-1 predictors block and gain/loss. For the standard task, the gain/loss variable and attained net scores remained the only significant predictor. For the reversed task, both expressive vocabulary and language group were additional significant predictors. Bilinguals showed fewer switches than their monolingual peers. For both the standard and the reversed task, model fits were improved in the second step.

### Research question 3: Mental effort in a hot executive function task

**FIGURE 3:**
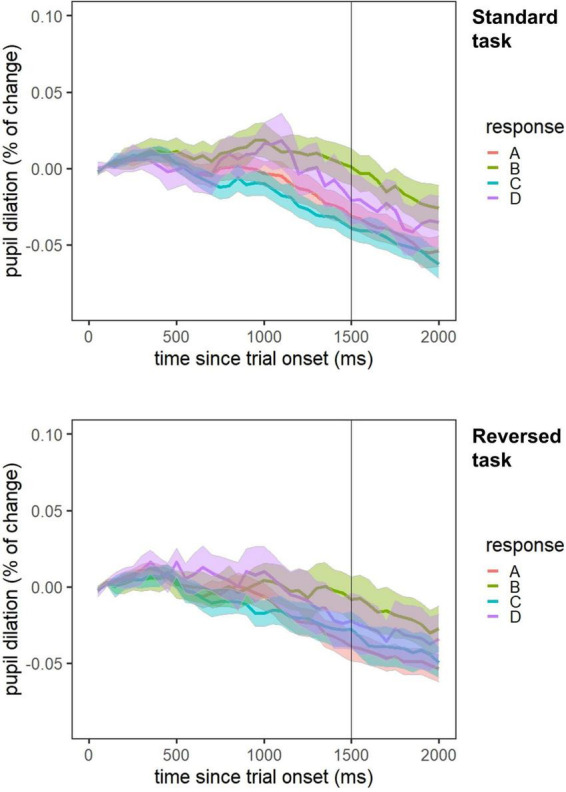
Time course of change in pupil dilation averaged within each door for both task versions. Change in pupil diameter in relation to baseline as a function of time (in milliseconds) from trial onset (with 95% confidence interval) is shown.

First, we tested a random intercept, fixed slope model and the two dummy coded variables specifying the selected door were added as predictors (Table 5). Intercepts indicate the mean percentage of changes in pupil dilation for door A. In both the standard and the reversed task, the dummy coded variable x_2_, indicating frequent vs. occasional gains/losses, reached statistical significance. Hence, mental effort for doors B and D differed significantly from door A (see also Figure 3).

**TABLE 5:** Multilevel models predicting change in pupil diameter from door chosen and language group.

	Standard task						Reversed task					
		
	Model 1			Model 2			Model 1			Model 2		
				
Variable	Estimate	SE	SD	Estimate	SE	SD	Estimate	SE	SD	Estimate	SE	SD
**Fixed effects**												
Intercept (door A)	0.00	0.01		0.00	0.02		0.00	0.01		0.00	0.02	
Door x_1_^a^ (advantageous vs. disadvantageous doors)	–0.01	0.01		–0.03	0.02		0.00	0.01		–0.00	0.02	
Door x_2_^b^ (frequent vs. occasional loss/gain)	0.03***	0.01		0.01	0.02		0.03***	0.01		0.04	0.02	
Door x_1_ × door x_2_	–0.01	0.01		0.02	0.03		–0.02	0.01		–0.04	0.03	
Language group^c^				–0.00	0.01					–0.00	0.01	
Door x_1_ × language group				0.01	0.01					0.00	0.01	
Door x_2_ × language group				0.01	0.01					–0.01	0.01	
Door x_1_ × door x_2_ × language group				–0.02	0.02					0.02	0.02	
**Random effects**												
Intercept			0.00			0.00			0.00			0.00
Residual			0.00			0.00			0.00			0.00
**Model fits**												
χ^2^ (*df*)				3.41(*4*)						1.67(*4*)		
AIC	–936.33			–931.74			–886.98			–880.65		
Pseudo-*R*^2^ ^d^	0.09			0.10			0.06			0.06		

^a^Dummy coded: 0 = doors A and B, 1 = doors C and D.

^b^Dummy coded: 0 = doors A and C, 1 = doors B and D.

^c^Dummy coded: monolingual = 0, bilingual = 1.

^d^Snijders and Boskers (1994).

***p < 0.001.

In the next step, in another random intercept, fixed slope model, language group and the respective interaction terms were added as additional predictors (Model 2, final model). For both tasks, none of the predictors reached statistical significance. For both the standard and the reversed task, model fits were not improved in the second step.

## Discussion

The aim of this study was to investigate performance in a “hot” EF task and allocation of mental effort in bilingual and monolingual elementary school children. In the past, several studies have found an advantage of bilingual versus monolingual individuals in tasks related to conflict monitoring (e.g., Kempert et al., 2011; Yang et al., 2011; Engel de Abreu et al., 2012; Kapa and Colombo, 2013; Poarch and Bialystok, 2015; Saalbach et al., 2016). A common basis seems to underlie performance on hot and cold EF tasks (e.g., Prencipe et al., 2011) and most everyday challenges draw on both cold and hot aspects of EF (Meuwissen and Zelazo, 2014). Moreover, the communicative challenges imposed by a bilingual environment might also draw on social cognitive skills (Yow and Markman, 2011, 2015) and these emotionally and motivationally relevant communicative situations might provide unique experiences for bilinguals to train their “cold” and “hot” EF in an integrated way. We hence expected bilinguals to show an advantage in a task of the “hot” EF domain *via* more mature decision-making as indicated by fewer switches. We further investigated whether bilinguals would show less mental effort during task execution than monolinguals.

In a first step, however, we investigated the development of monolingual and bilingual children’s decision-making strategies over the course of the task. In the standard version of the IGT, decision-making did not improve significantly over the course of the task in both groups of children. In the reversed task version, children chose more advantageous doors over the course of the task. This decision-making behavior did not differ between monolinguals and bilinguals (Research aim 1). In both versions of the task, children switched doors more often after having faced a loss. This was true for both language groups. In the reversed task version, bilinguals switched their choices less often than monolinguals, independent of gain and loss trials (Research aim 2). In addition, by controlling for net scores, we examined how switching behavior was related to the selection of advantageous versus disadvantageous doors. The frequency of switches was positively related to the net scores in the standard task, more switches hence resulted in higher net scores. In the reversed task, the frequency of switches was negatively related to the net scores. Concerning mental effort, we did not find significant differences between monolinguals and bilinguals. Differences of effort allocation were present between the four different doors (Research aim 3).

The finding that children in the reversed task learned more rapidly to select advantageous doors is in line with past research (Bechara et al., 2000; Crone et al., 2005). Winning items might affect children’s decision-making more than losing items (Huizenga et al., 2007), and children might be guided by immediate gains while ignoring future prospects (Schlottmann, 2000). That is, in the standard task, children kept on choosing door B, because it provided the highest gains in 90% of the trials. In the reversed task children might have ignored the higher number of lost gems in doors A and B while focusing on the possibility of gaining higher amounts in these two options at some point. Children with better syntactical knowledge chose on average less advantageous doors in the reversed task. This was a surprising result. However, given that performance in traditional EF tasks is related to linguistic abilities (Bohlmann et al., 2015; Cadima et al., 2019), it might be another indicator that decision-making as one example of hot EF seems to be related to different cognitive processes than EF operating in emotionally neutral settings. The decision-making behavior did not differ between monolinguals and bilinguals. The fact, however, that decision-making did improve over the course of the task, but only marginally in comparison to older children (Crone and van der Molen, 2004), points to the possibility that the hypothesized differences between monolinguals and bilinguals guided by more mature switching behavior could appear in later childhood and not as early as in elementary school.

In the past, research has reported bilingual advantages in the domain of cold EF, especially in inhibitory control tasks (e.g., Kempert et al., 2011; Yang et al., 2011; Engel de Abreu et al., 2012; Poarch and van Hell, 2012; Kapa and Colombo, 2013; Poarch and Bialystok, 2015; Saalbach et al., 2016). Since cold and hot EF appear to be related to a common cognitive basis (e.g., Prencipe et al., 2011), we had expected that bilinguals might be advantaged in a hot EF task by more mature decision-making as indicated by less explorative behavior and hence fewer switches. This effect appeared only in the reversed task of the IGT and it was not very pronounced. Several explanations for this finding seem plausible. First, several studies have shown that substantial improvements in favorable decision-making might appear only in later childhood. In one of the first studies contrasting performance in the IGT in several age groups (Crone and van der Molen, 2004), adolescents aged 13–15 years significantly outperformed two groups of younger children aged 6–9 and 10–12 years, while differences between those two younger groups of children were not apparent. Similarly, Prencipe et al. (2011) observed improvements in IGT performance only for children aged 14–15 years in comparison to three younger groups between the ages of 8–13. Thus, possible improvements in switching behavior and accompanying differences between monolingual and bilingual children might likewise appear only in adolescence. However, because EF development typically continues beyond the preschool years and significant improvements appear during the school years (Best et al., 2009), because the elementary school is a critical period for EF supporting academic learning (e.g., Cirino et al., 2018) and because studies on hot EF performance in bilingual children are still scarce in this age group, we had decided to focus on younger instead of older school-children in the current study. Second, the question of whether hot and cold measures of executive function represent one common or distinct processes is still being discussed. Even though both hot and cool EF operate in situations requiring conscious and goal-directed behavior, studies including behavioral data diverge regarding conclusions on the processes’ distinctiveness. While some studies find one common underlying EF factor (e.g., Prencipe et al., 2011), others report a two-factor structure (Carlson and Meltzoff, 2008; Montroy et al., 2019). Furthermore, studies differ with regard to relations between measures of the cold EF domain and the IGT. In a large adult sample, Gansler et al. (2011b) used structural equation modeling to detect related neuropsychological processes. They found that general intelligence and attention were related to IGT performance, but EF ability to a lesser extent. In a more recent study, Ouerchefani et al. (2019) investigated correlations between IGT performance and classical EF measures and found that performance was related to indices of inhibition, cognitive flexibility and planning. In a recent review on the functional organization of cold versus hot EF in the brain, Salehinejad et al. (2021) describe how distinct brain circuits might be involved in the organization of the respective processes. While the lateral PFC and dorsal ACC are more involved in cold EF, the medial–orbital PFC, ventral ACC, and posterior cingulate cortex (PCC) are related to hot EF. The authors stress, however, that this distinction depends on factors such as task features and context and that the identified regions are not functionally limited to cold or hot cognition. To sum up, there is some evidence that hot and cold EF share a common basis but are nonetheless distinguishable constructs. Therefore, results favoring bilinguals over monolinguals in tasks of the cold EF domain might not be readily transferable to EF operating in emotional relevant settings. Lastly, two recent meta-analyses (Gunnerud et al., 2020; Lowe et al., 2021) again question the robustness of general EF performance differences between monolingual and bilingual children. Lowe et al. (2021) identified response inhibition, including studies administering go/no go or stop-signal tasks, as the only EF domain favoring bilinguals over monolinguals. Gunnerud et al. (2020) found a bilingual advantage for cold inhibition (by others referred to as conflict resolution tasks), switching and monitoring, but point out that monitoring and inhibition were affected by publication bias. Hence, if performance differences between monolinguals and bilinguals exist, they might be hard to detect and be dependent on several context factors (Morton, 2015; Paap et al., 2015). In the case of hot EF, they might also be overshadowed by other operating processes.

In our analyses, switching behavior was related to performance in the IGT as indicated *via* net scores. Past research has shown that adults’ more mature decision-making is guided by fewer switches in the IGT (Cassotti et al., 2014). Indeed, the less children switched doors in the reversed task the higher net scores they attained. Apparently, better average performance in the reversed task might have resulted from children inhibiting intuitive exploration after having discovered a door that provided relatively high or frequent occasional gains in addition to regular losses. This result extends past research with adults (Aïte et al., 2012). In the standard task, however, more switches were related to higher net scores. Presumably, children who switched less often in this task version stuck with the disadvantageous door B without exploring more favorable options. Hence, children of this age seem to still lack the cognitive flexibility to adjust their selection strategies, at least if future losses are not immediately apparent. Exploration during the first blocks of the IGT seems to be necessary to gain an overview of the task’s prerequisites. Some researchers already suggested to adapt the conventional IGT metrics by differentiating different task phases and deck selections metrics to improve the usefulness of the test (Gansler et al., 2011a, b).

Our last research aim was to investigate the exhibited mental effort during IGT performance. No differences between monolinguals and bilinguals were apparent. We found, however, differences in pupil dilation between the doors subjects could choose. Doors B and D produced significantly more effort in comparison to the baseline door A. Those two options are related to only occasional punishment compared to the other options where 50% of trials result in losing points. Pupillary changes during IGT performance have been interpreted in terms of measuring surprise during negative feedback presentation (Lavín et al., 2013). The pupil dilates in reaction to brain activity in areas like the ACC. The ACC in turn operates in the context of conflict detection that can operate as a teaching signal (Botvinick, 2007). The pupillary changes in the IGT could thus be an indicator of a learning process taking place during the response phase of surprising (because being only occasional) outcomes. In a previous study, Enke et al. (2022) found differences in effort allocation between monolinguals and bilinguals solving a planning task. Possibly, different processes are involved in the execution of a complex, problem-based task requiring several conscious and well thought steps in comparison to a decision-making task that can be solved by selecting options in a more intuitive and automatized manner. This has already been shown by studies identifying differing brain regions and circuits related to performance of hot vs. cold EF tasks (Happaney et al., 2004; Salehinejad et al., 2021). The findings observing a higher processing efficiency in bilinguals might hence be limited to the execution of traditional, cold EF tasks.

### Limitations

The present study adds to the literature by investigating performance and related processes in a hot EF task in monolingual and bilingual school children. Even though we did not find substantial group differences, future research should expand our approach by including other tasks of the hot EF domain. As Zelazo and Carlson (2020) argue, the IGT might depend on both hot and cold EF skills. Thus, measures like delay of gratification or delay discounting should also be investigated to provide a full picture of hot EF in bilingual children.

### Conclusion

Our study is the first to investigate a well-established measure of the so-called “hot” EF domain in a sample of monolingual and bilingual elementary school children. Except for minor effects, we did not observe substantial differences between the two groups of children, not concerning overall performance, nor in underlying cognitive processes. We conclude that bilinguals’ EF performance advantages and advantages found in terms of processing efficiency might be limited to tasks measuring traditional, cold EF.

## Data availability statement

The raw data supporting the conclusions of this article will be made available by the authors, without undue reservation.

## Ethics statement

The studies involving human participants were reviewed and approved by Leipzig University’s Ethics Committee. Written informed consent to participate in this study was provided by the participants or their legal guardian/next of kin.

## Author contributions

SE, CG, JK, and HS contributed to the conception and design of the study. SE and JK organized the database. SE performed the statistical analysis and wrote the first draft of the manuscript. All authors contributed to the manuscript revision, read, and approved the submitted version.

## Footnotes

1 Like in the original task, the number of lost (standard task) or gained (reversed task) gems for door A varied. It was set to either 8 (once in ten trials), 10 (three times in ten trials) or 12 (once in ten trials). This resulted in a loss/gain of 10 gems on average.

2 Like in the original task, the number of lost (standard task) or gained gems (reversed task) for door C varied. It was set to either 1 (once in ten trials), 2 (three times in ten trials) or 3 (once in ten trials). This resulted in a loss/gain of 2 gems on average.

3 In each condition, a trial was counted as a gain (standard task) or loss (reversed task), when the number of gained/lost gems was greater than the number of lost/gained gems. Thus, when the number of gained and lost gems was equal, it was counted as a loss in the standard version of the task and as a gain in the reversed version of the task.

## References

[B1] Abutalebi J. Della Rosa P. A. Green D. W. Hernandez M. Scifo P. Keim R. (2012). Bilingualism tunes the anterior cingulate cortex for conflict monitoring. *Cerebral Cortex* 22 2076–2086. 10.1093/cercor/bhr287 22038906

[B2] Adesope O. O. Lavin T. Thompson T. Ungerleider C. (2010). A systematic review and meta-analysis of the cognitive correlates of bilingualism. *Rev. Educ. Res.* 80 207–245. 10.3102/0034654310368803

[B3] Aïte A. Cassotti M. Rossi S. Poirel N. Lubin A. Houdé O. (2012). Is human decision making under ambiguity guided by loss frequency regardless of the costs? A developmental study using the soochow gambling task. *J. Exp. Child Psychol.* 113 286–294. 10.1016/j.jecp.2012.05.008 22727674

[B4] Antoniou M. (2019). The advantages of bilingualism debate. *Ann. Rev. Ling.* 5 395–415. 10.1146/annurev-linguistics-011718-011820

[B5] Aston-Jones G. Cohen J. D. (2005). An integrative theory of locus coeruleus-norepinephrine function: Adaptive gain and optimal performance. *Ann. Rev. Neurosci.* 28 403–450. 10.1146/annurev.neuro.28.061604.135709 16022602

[B6] Bagneux V. Thomassin N. Gonthier C. Roulin J. (2013). Working memory in the processing of the iowa gambling task: an individual differences approach. *PLoS One* 8:e81498. 10.1371/journal.pone.0081498 24278447 PMC3835610

[B7] Barac R. Bialystok E. Castro D. C. Sanchez M. (2014). The cognitive development of young dual language learners: A critical review. *Early Child. Res. Quart.* 29 699–714. 10.1016/j.ecresq.2014.02.003 25284958 PMC4180217

[B8] Barker R. M. Bialystok E. (2019). Processing differences between monolingual and bilingual young adults on an emotion n-back task. *Brain Cogn.* 134 29–43. 10.1016/j.bandc.2019.05.004 31108367 PMC6556413

[B9] Bates D. Mächler M. Bolker B. Walker S. (2015). Fitting linear mixed-effects models using lme4. *J. Statist. Software* 67:i01. 10.18637/jss.v067.i01

[B10] Beatty J. Lucero-Wagoner B. (2000). “The pupillary system,” in *Handbook of Psychophysiology*, 2nd Edn, eds Cacioppo J. T. Tassinary L. G. Berntson G. G. (Cambridge University Press), 142–162.

[B11] Bechara A. Damasio A. R. Damasio H. Anderson S. W. (1994). Insensitivity to future consequences following damage to human prefrontal cortex. *Cognition* 50 7–15. 10.1016/0010-0277(94)90018-3 8039375

[B12] Bechara A. Damasio H. Damasio A. R. (2000). Emotion, decision making and the orbitofrontal cortex. *Cerebral Cortex* 10 295–307. 10.1093/cercor/10.3.295 10731224

[B13] Becker T. M. Prat C. S. Stocco A. (2016). A network-level analysis of cognitive flexibility reveals a differential influence of the anterior cingulate cortex in bilinguals versus monolinguals. *Neuropsychologia* 85 62–73. 10.1016/j.neuropsychologia.2016.01.020 26796713

[B14] Berroir P. Ghazi-Saidi L. Dash T. Adrover-Roig D. Benali H. Ansaldo A. I. (2017). Interference control at the response level: Functional networks reveal higher efficiency in the bilingual brain. *J. Neurolin.* 43 4–16. 10.1016/j.jneuroling.2016.09.007

[B15] Best J. R. Miller P. H. Jones L. L. (2009). Executive functions after age 5: Changes and correlates. *Dev. Rev.* 29 180–200. 10.1016/j.dr.2009.05.002 20161467 PMC2792574

[B16] Bialystok E. (2017). The bilingual adaptation: How minds accommodate experience. *Psychol. Bull.* 143 233–262. 10.1037/bul0000099 28230411 PMC5324728

[B17] Bialystok E. Craik F. I. M. Green D. W. Gollan T. H. (2009). Bilingual minds. *Psychol. Sci. Public Int. J. Am. Psychol. Soc.* 10 89–129. 10.1177/1529100610387084 26168404

[B18] Bohlmann N. L. Maier M. F. Palacios N. (2015). Bidirectionality in self-regulation and expressive vocabulary: Comparisons between monolingual and dual language learners in preschool. *Child Dev.* 86 1094–1111. 10.1111/cdev.12375 25906925

[B19] Bonmassar C. Widmann A. Wetzel N. (2020). The impact of novelty and emotion on attention-related neuronal and pupil responses in children. *Dev. Cogn. Neurosci.* 42:100766. 10.1016/j.dcn.2020.100766 32452459 PMC7068055

[B20] Botvinick M. M. (2007). Conflict monitoring and decision making: Reconciling two perspectives on anterior cingulate function. *Cogn. Affect. Behav. Neurosci.* 7 356–366. 10.3758/cabn.7.4.356 18189009

[B21] Botvinick M. M. Braver T. S. Barch D. M. Carter C. S. Cohen J. D. (2001). Conflict monitoring and cognitive control. *Psychol. Rev.* 108 624–652. 10.1037/0033-295X.108.3.624 11488380

[B22] Brock L. L. Rimm-Kaufman S. E. Nathanson L. Grimm K. J. (2009). The contributions of ‘hot’ and ‘cool’ executive function to children’s academic achievement, learning-related behaviors, and engagement in kindergarten. *Early Child. Res. Quart.* 24 337–349. 10.1016/j.ecresq.2009.06.001

[B23] Buelow M. T. Suhr J. A. (2009). Construct validity of the Iowa gambling task. *Neuropsychol. Rev.* 19 102–114. 10.1007/s11065-009-9083-4 19194801

[B24] Cadima J. Barros S. Ferreira T. Serra-Lemos M. Leal T. Verschueren K. (2019). Bidirectional associations between vocabulary and self-regulation in preschool and their interplay with teacher–child closeness and autonomy support. *Early Child. Res. Quart.* 46 75–86. 10.1016/j.ecresq.2018.04.004

[B25] Carlson S. M. Meltzoff A. N. (2008). Bilingual experience and executive functioning in young children. *Dev. Sci.* 11 282–298. 10.1111/j.1467-7687.2008.00675.x 18333982 PMC3647884

[B26] Cassotti M. Aïte A. Osmont A. Houdé O. Borst G. (2014). What have we learned about the processes involved in the Iowa gambling task from developmental studies? *Front. Psychol.* 5:915. 10.3389/fpsyg.2014.00915 25191295 PMC4138612

[B27] Castillo A. (2021). *The Bilingual Advantage Debate: Are We Getting Warmer? Ph. D, Thesis.* University of California, Merced.

[B28] Cirino P. T. Ahmed Y. Miciak J. Taylor W. P. Gerst E. H. Barnes M. A. (2018). A framework for executive function in the late elementary years. *Neuropsychology* 32 176–189. 10.1037/neu0000427 29528682 PMC5851451

[B29] Core Team R. (2021). *R: A Language and Environment for Statistical Computing [Computer Software].* Vienna, Austria: R Foundation for Statistical Computing.

[B30] Crone E. A. van der Molen M. W. (2004). Developmental changes in real life decision making: Performance on a gambling task previously shown to depend on the ventromedial prefrontal cortex. *Dev. Neuropsychol.* 25 251–279. 15147999 10.1207/s15326942dn2503_2

[B31] Crone E. A. Bunge S. A. Latenstein H. van der Molen M. W. (2005). Characterization of children’s decision making: Sensitivity to punishment frequency, not task complexity. *Child Neuropsychol.* 11 245–263. 10.1080/092970490911261 16036450

[B32] Cui J. F. Wang Y. Shi H. S. Liu L. L. Chen X. J. Chen Y. H. (2015). Effects of working memory load on uncertain decision-making: Evidence from the Iowa gambling task. *Front. Psychol.* 6:162. 10.3389/fpsyg.2015.00162 25745409 PMC4333774

[B33] Della Rosa P. A. Videsott G. Borsa V. M. Canini M. Weekes B. S. Franceschini R. (2013). A neural interactive location for multilingual talent. *Cortex* 49 605–608. 10.1016/j.cortex.2012.12.001 23294573

[B34] Eckstein M. K. Guerra-Carrillo B. Miller Singley A. T. Bunge S. A. (2017). Beyond eye gaze: What else can eyetracking reveal about cognition and cognitive development? *Dev. Cogn. Neurosci.* 25 69–91. 10.1016/j.dcn.2016.11.001 27908561 PMC6987826

[B35] Enders C. K. Tofighi D. (2007). Centering predictor variables in cross-sectional multilevel models: A new look at an old issue. *Psychol. Methods* 12, 121–138. 10.1037/1082-989x.12.2.121 17563168

[B36] Engel de Abreu P. M. J. (2011). Working memory in multilingual children: Is there a bilingual effect? *Memory* 19 529–537. 10.1080/09658211.2011.590504 21864216

[B37] Engel de Abreu P. M. J. Cruz-Santos A. Tourinho C. J. Martin R. Bialystok E. (2012). Bilingualism enriches the poor: Enhanced cognitive control in low-income minority children. *Psychol. Sci.* 23 1364–1371. 10.1177/0956797612443836 23044796 PMC4070309

[B38] Enke S. Gunzenhauser C. Hepach R. Karbach J. Saalbach H. (2022). Differences in cognitive processing? The role of verbal processes and mental effort in bilingual and monolingual children’s planning performance. *J. Exp. Child Psychol.* 213:105255. 10.1016/j.jecp.2021.105255 34388641

[B39] Fox A. V. (2013). *TROG-D: Test Zur Überprüfung Des Grammatikverständnisses*, 6th Edn. Idstein, Germany: Schulz-Kirchner.

[B40] Gansler D. A. Jerram M. W. Vannorsdall T. D. Schretlen D. J. (2011a). Comparing alternative metrics to assess performance on the Iowa gambling task. *J. Clin. Exp. Neuropsychol.* 33 1040–1048. 10.1080/13803395.2011.596820 21916658

[B41] Gansler D. A. Jerram M. W. Vannorsdall T. D. Schretlen D. J. (2011b). Does the Iowa gambling task measure executive function? *Arch. Clin. Neuropsychol.* 26 706–717. 10.1093/arclin/acr082 22015855 PMC3254153

[B42] Garon N. Bryson S. E. Smith I. M. (2008). Executive function in preschoolers: A review using an integrative framework. *Psychol. Bull.* 134 31–60. 10.1037/0033-2909.134.1.31 18193994

[B43] Glück C. W. (2011). *WWT 6–10: Wortschatz- und Wortfindungstest für 6- bis 10-Jährige*, 2nd Edn. Jena, Germany: Urban & Fischer.

[B44] Gold B. T. (2015). Executive control, brain aging and bilingualism. *Cortex* 73 369–370. 10.1016/j.cortex.2015.06.014 26189683

[B45] Gold B. T. Kim C. Johnson N. F. Kryscio R. J. Smith C. D. (2013). Lifelong bilingualism maintains neural efficiency for cognitive control in aging. *J. Neurosci.* 33 387–396. 10.1523/JNEUROSCI.3837-12.2013 23303919 PMC3710134

[B46] Grady C. L. Luk G. Craik F. I. M. Bialystok E. (2015). Brain network activity in monolingual and bilingual older adults. *Neuropsychologia* 66 170–181. 10.1016/j.neuropsychologia.2014.10.042 25445783 PMC4898959

[B47] Green D. W. (1998). Mental control of the bilingual lexico-semantic system. *Biling. Lang. Cogn.* 1 67–81. 10.1017/S1366728998000133

[B48] Green D. W. Abutalebi J. (2013). Language control in bilinguals: The adaptive control hypothesis. *J. Cogn. Psychol.* 25 515–530. 10.1080/20445911.2013.796377 25077013 PMC4095950

[B49] Gunnerud H. L. ten Braak D. Reikerås E. K. L. Donolato E. Melby-Lervåg M. (2020). Is bilingualism related to a cognitive advantage in children? A systematic review and meta-analysis. *Psychol. Bull.* 146 1059–1083. 10.1037/bul0000301 32914991

[B50] Hakuta K. (1986). *Mirror of Language: The Debate on Bilingualism.* New York, NY: Basic Books.

[B51] Happaney K. Zelazo P. D. Stuss D. T. (2004). Development of orbitofrontal function: Current themes and future directions. *Brain Cogn.* 55 1–10. 10.1016/j.bandc.2004.01.001 15134839

[B52] Hepach R. Vaish A. Tomasello M. (2012). Young children are intrinsically motivated to see others helped. *Psychol. Sci.* 23 967–972. 10.1177/0956797612440571 22851443

[B53] Hofmann W. Schmeichel B. J. Baddeley A. D. (2012). Executive functions and self-regulation. *Trends Cogn. Sci.* 16 174–180. 10.1016/j.tics.2012.01.006 22336729

[B54] Hooper C. J. Luciana M. Conklin H. M. Yarger R. S. (2004). Adolescents’ performance on the Iowa gambling task: Implications for the development of decision making and ventromedial prefrontal cortex. *Dev. Psychol.* 40 1148–1158. 10.1037/0012-1649.40.6.1148 15535763

[B55] Huizenga H. M. Crone E. A. Jansen B. J. (2007). Decision-making in healthy children, adolescents and adults explained by the use of increasingly complex proportional reasoning rules. *Dev. Sci.* 10 814–825. 10.1111/j.1467-7687.2007.00621.x 17973798

[B56] Hülsheger U. R. Lang J. W. B. Depenbrock F. Fehrmann C. Zijlstra F. R. H. Alberts H. J. E. M. (2014). The power of presence: The role of mindfulness at work for daily levels and change trajectories of psychological detachment and sleep quality. *J. Appl. Psychol.* 99, 1113–1128. 10.1037/a0037702 25198098

[B57] Janus M. Bialystok E. (2018). Working memory with emotional distraction in monolingual and bilingual children. *Front. Psychol.* 9:1582. 10.3389/fpsyg.2018.01582 30210408 PMC6120977

[B58] Just M. A. Carpenter P. A. Miyake A. (2003). Neuroindices of cognitive workload: Neuroimaging, pupillometric and event-related potential studies of brain work. *Theor. Issues Ergon. Sci.* 4 56–88. 10.1080/14639220210159735

[B59] Kapa L. L. Colombo J. (2013). Attentional control in early and later bilingual children. *Cogn. Dev.* 28 233–246. 10.1016/j.cogdev.2013.01.011 24910499 PMC4044912

[B60] Karbach J. Unger K. (2014). Executive control training from middle childhood to adolescence. *Front. Psychol.* 5:390. 10.3389/fpsyg.2014.00390 24847294 PMC4019883

[B61] Kelly A. M. C. Garavan H. (2005). Human functional neuroimaging of brain changes associated with practice. *Cerebral Cortex* 15 1089–1102. 10.1093/cercor/bhi005 15616134

[B62] Kempert S. Saalbach H. Hardy I. (2011). Cognitive benefits and costs of bilingualism in elementary school students: The case of mathematical word problems. *J. Educ. Psychol.* 103 547–561. 10.1037/a0023619

[B63] Kerns J. G. Cohen J. D. MacDonald A. W. Cho R. Y. Stenger V. A. Carter C. S. (2004). Anterior cingulate conflict monitoring and adjustments in control. *Science* 303 1023–1026. 10.1126/science.1089910 14963333

[B64] Kerr A. Zelazo P. D. (2004). Development of “hot” executive function: The children’s gambling task. *Brain Cogn.* 55 148–157. 10.1016/S0278-2626(03)00275-6 15134849

[B65] Lavín C. San Martín R. Rosales Jubal E. (2013). Pupil dilation signals uncertainty and surprise in a learning gambling task. *Front. Behav. Neurosci.* 7:218. 10.3389/fnbeh.2013.00218 24427126 PMC3879532

[B66] Li P. Legault J. Litcofsky K. A. (2014). Neuroplasticity as a function of second language learning: Anatomical changes in the human brain. *Cortex* 58 301–324. 10.1016/j.cortex.2014.05.001 24996640

[B67] Lowe C. J. Cho I. Goldsmith S. F. Morton J. B. (2021). The bilingual advantage in children’s executive functioning is not related to language status: A meta-analytic review. *Psychol. Sci.* 32 1115–1146. 10.1177/0956797621993108 34213379 PMC8641133

[B68] Mathôt S. Schreij D. Theeuwes J. (2012). OpenSesame: An open-source, graphical experiment builder for the social sciences. *Behav. Res. Methods* 44 314–324. 22083660 10.3758/s13428-011-0168-7 PMC3356517

[B69] McClelland M. M. Cameron C. E. (2012). Self-regulation in early childhood: Improving conceptual clarity and developing ecologically valid measures. *Child Dev. Perspect.* 6 136–142. 10.1111/j.1750-8606.2011.00191.x

[B70] McGlamery M. E. Ball S. E. Henley T. B. Besozzi M. (2007). Theory of mind, attention, and executive function in kindergarten boys. *Emot. Behav. Diff.* 12 29–47. 10.1080/13632750601135899

[B71] McNeish D. M. Stapleton L. M. (2016). The effect of small sample size on two-level model estimates: A review and illustration. *Educ. Psychol. Rev.* 28 295–314. 10.1007/s10648-014-9287-x

[B72] Mechelli A. Crinion J. T. Noppeney U. O’Doherty J. Ashburner J. Frackowiak R. S. (2004). Structural plasticity in the bilingual brain. *Nature* 431:757. 10.1038/431757a 15483594

[B73] Meuwissen A. S. Zelazo P. D. (2014). Hot and cool executive function: Foundations for learning and healthy development. *Zero Three* 35 18–23.

[B74] Montroy J. J. Merz E. C. Williams J. M. Landry S. H. Johnson U. Y. Zucker T. A. (2019). Hot and cool dimensionality of executive function: Model invariance across age and maternal education in preschool children. *Early Child. Res. Quart.* 49 188–201. 10.1016/j.ecresq.2019.06.011

[B75] Morton J. B. (2015). Still waiting for real answers. *Cortex* 73 352–353. 10.1016/j.cortex.2015.07.010 26298268

[B76] Nayak S. Tarullo A. R. (2020). Error-related negativity (ERN) and ‘hot’ executive function in bilingual and monolingual preschoolers. *Biling. Lang. Cogn.* 23 897–908. 10.1017/S1366728919000725

[B77] O’Toole S. Monks C. P. Tsermentseli S. (2018). Associations between and development of cool and hot executive functions across early childhood. *Br. J. Dev. Psychol.* 36 142–148. 10.1111/bjdp.12226 29226486

[B78] Ouerchefani R. Ouerchefani N. Allain P. Ben Rejeb M. R. Le Gall D. (2019). Relationships between executive function, working memory, and decision-making on the Iowa gambling task: Evidence from ventromedial patients, dorsolateral patients, and normal subjects. *J. Neuropsychol.* 13 432–461. 10.1111/jnp.12156 29667317

[B79] Paap K. R. Anders-Jefferson R. Mikulinsky R. Masuda S. Mason L. (2019). On the encapsulation of bilingual language control. *J. Memory Lang.* 105 76–92. 10.1016/j.jml.2018.12.001

[B80] Paap K. R. Johnson H. A. Sawi O. (2015). Bilingual advantages in executive functioning either do not exist or are restricted to very specific and undetermined circumstances. *Cortex* 69 265–278. 10.1016/j.cortex.2015.04.014 26048659

[B81] Paap K. R. Johnson H. A. Sawi O. (2016). Should the search for bilingual advantages in executive functioning continue? *Cortex* 74 305–314. 10.1016/j.cortex.2015.09.010 26586100

[B82] Peal E. Lambert W. E. (1962). The relation of bilingualism to intelligence. *Psychol. Monog.* 76 1–23.

[B83] Peterson E. Welsh M. C. (2014). “The development of hot and cool executive functions in childhood and adolescence: Are we getting warmer?,” in *Handbook of Executive Functioning*, eds Goldstein S. Naglieri J. A. (Springer), 45–65. 10.1007/978-1-4614-8106-5_4

[B84] Pliatsikas C. (2020). Understanding structural plasticity in the bilingual brain: The dynamic restructuring model. *Biling. Lang. Cogn.* 23 459–471. 10.1017/S1366728919000130

[B85] Poarch G. J. Bialystok E. (2015). Bilingualism as a model for multitasking. *Dev. Rev.* 35 113–124. 10.1016/j.dr.2014.12.003 25821336 PMC4371212

[B86] Poarch G. J. van Hell J. G. (2012). Executive functions and inhibitory control in multilingual children: Evidence from second-language learners, bilinguals, and trilinguals. *J. Exp. Child Psychol.* 113 535–551. 10.1016/j.jecp.2012.06.013 22892367

[B87] Poulin-Dubois D. Blaye A. Coutya J. Bialystok E. (2011). The effects of bilingualism on toddlers’ executive functioning. *J. Exp. Child Psychol.* 108 567–579. 10.1016/j.jecp.2010.10.009 21122877 PMC4346342

[B88] Prencipe A. Kesek A. Cohen J. Lamm C. Lewis M. D. Zelazo P. D. (2011). Development of hot and cool executive function during the transition to adolescence. *J. Exp. Child Psychol.* 108 621–637. 10.1016/j.jecp.2010.09.008 21044790

[B89] Quené H. van den Bergh H. (2004). On multi-level modeling of data from repeated measures designs: a tutorial. *Speech Commun.* 43 103–121. 10.1016/j.specom.2004.02.004

[B90] Raven J. Raven J. C. Court J. H. (1998). *Manual for Raven’s Progressive Matrices and Vocabulary Scales, Section 2: The Coloured Progressive Matrices.* Oxford, UK: Oxford Psychologists Press.

[B91] Saalbach H. Gunzenhauser C. Kempert S. Karbach J. (2016). Der einfluss von mehrsprachigkeit auf mathematische fähigkeiten bei grundschulkindern mit niedrigem sozioökonomischen status. *Frühe Bildung* 5 73–81. 10.1026/2191-9186/a000255

[B92] Saer D. J. (1923). The effects of bilingualism on intelligence. *Br. J. Psychol.* 14 25–38.

[B93] Salehinejad M. A. Ghanavati E. Rashid M. H. A. Nitsche M. A. (2021). Hot and cold executive functions in the brain: A prefrontal-cingular network. *Brain Neurosci. Adv.* 5:23982128211007769. 10.31219/osf.io/5sghe PMC8076773 33997292

[B94] Schlottmann A. (2000). Children’s judgements of gambles: A disordinal violation of utility. *J. Behav. Dec. Make.* 13 77–89. 10.1002/(SICI)1099-0771(200001/03)13:1<77::AID-BDM344<3.0.CO;2-Y

[B95] Shallice T. (1982). Specific impairments of planning. *Philos. Trans. R. Soc. London Seri. B Sci.* 298 199–209. 10.1098/rstb.1982.0082 6125971

[B96] Snijders T. A. B. Boskers R. J. (1994). Modeled variance in two-level models. *Sociol. Methods Res.* 22 342–363. 10.1177/0049124194022003004

[B97] Sulpizio S. Del Maschio N. Fedeli D. Abutalebi J. (2020). Bilingual language processing: A meta-analysis of functional neuroimaging studies. *Neurosci. Biobehav. Rev.* 108 834–853. 10.1016/j.neubiorev.2019.12.014 31838193

[B98] Titz C. Karbach J. (2014). Working memory and executive functions: Effects of training on academic achievement. *Psychol. Res.* 78 852–868. 10.1007/s00426-013-0537-1 24389706

[B99] Toplak M. E. Sorge G. B. Benoit A. West R. F. Stanovich K. E. (2010). Decision-making and cognitive abilities: A review of associations between Iowa gambling task performance, executive functions, and intelligence. *Clin. Psychol. Rev.* 30 562–581. 10.1016/j.cpr.2010.04.002 20457481

[B100] Valian V. (2015). Bilingualism and cognition: A focus on mechanisms. *Biling. Lang. Cogn.* 18 47–50. 10.1017/S1366728914000698

[B101] van der Wel P. van Steenbergen H. (2018). Pupil dilation as an index of effort in cognitive control tasks: A review. *Psychon. Bull. Rev.* 25 2005–2015. 10.3758/s13423-018-1432-y 29435963 PMC6267528

[B102] Vaughn K. A. Greene M. R. Ramos Nuñez A. I. Hernandez A. E. (2015). The importance of neuroscience in understanding bilingual cognitive control. *Cortex* 73 373–374. 10.1016/j.cortex.2015.06.010 26211434 PMC5938746

[B103] Verhagen J. Mulder H. Leseman P. P. M. (2017). Effects of home language environment on inhibitory control in bilingual three-year-old children. *Biling. Lang. Cogn.* 20 114–127. 10.1017/S1366728915000590

[B104] von Bastian C. C. Souza A. S. Gade M. (2016). No evidence for bilingual cognitive advantages: A test of four hypotheses. *J. Exp. Psychol. General* 145 246–258. 10.1037/xge0000120 26523426

[B105] Welsh M. Peterson E. (2014). Issues in the conceptualization and assessment of hot executive functions in childhood. *J. Int. Neuropsychol. Soc.* 20 152–156. 10.1017/S1355617713001379 24468077

[B106] White L. J. Alexander A. Greenfield D. B. (2017). The relationship between executive functioning and language: Examining vocabulary, syntax, and language learning in preschoolers attending head start. *J. Exp. Child Psychol.* 164 16–31. 10.1016/j.jecp.2017.06.010 28772245

[B107] Yang S. Yang H. Lust B. (2011). Early childhood bilingualism leads to advances in executive attention: Dissociating culture and language. *Biling. Lang. Cogn.* 14 412–422. 10.1017/S1366728910000611

[B108] Yeung N. Botvinick M. M. Cohen J. D. (2004). The neural basis of error detection: Conflict monitoring and the error-related negativity. *Psychol. Rev.* 111 931–959. 15482068 10.1037/0033-295x.111.4.939

[B109] Yow W. Q. Markman E. M. (2011). Young bilingual children’s heightened sensitivity to referential cues. *J. Cogn. Dev.* 12 12–31. 10.1080/15248372.2011.539524

[B110] Yow W. Q. Markman E. M. (2015). A bilingual advantage in how children integrate multiple cues to understand a speaker’s referential intent. *Biling. Lang. Cogn.* 18 391–399. 10.1017/S1366728914000133

[B111] Zelazo P. D. Carlson S. M. (2012). Hot and cool executive function in childhood and adolescence: Development and plasticity. *Child Dev. Perspect.* 47 354–360. 10.1111/j.1750-8606.2012.00246.x

[B112] Zelazo P. D. Carlson S. M. (2020). The neurodevelopment of executive function skills: Implications for academic achievement gaps. *Psychol. Neurosci.* 13 273–298. 10.1037/pne0000208

[B113] Zelazo P. D. Müller U. (2002). “Executive function in typical and atypical development,” in *Handbook of Childhood Cognitive Development*, ed. Goswami U. C. (Blackwell Publishers), 445–469.

[B114] Zelazo P. D. Craik F. I. M. Booth L. (2004). Executive function across the life span. *Acta Psychol.* 115 167–183. 10.1016/j.actpsy.2003.12.005 14962399

